# Surgical Management of Primary Anorectal Melanoma: Is Less More?

**DOI:** 10.1007/s12029-023-01009-z

**Published:** 2024-01-05

**Authors:** Michael G. Fadel, Hesham S. Mohamed, Justin Weir, Andrew J. Hayes, James Larkin, Myles J. Smith

**Affiliations:** 1 The Sarcoma, Melanoma and Rare Tumours Unit, The Royal Marsden Hospital NHS Foundation Trust, London, UK; 2 Department of Surgery and Cancer, Imperial College London, London, UK; 3 The Institute of Cancer Research, The Royal Marsden Hospital NHS Foundation Trust, London, UK; 4 Department of Cellular Pathology, The Royal Marsden Hospital NHS Foundation Trust, London, UK; 5 Department of Medical Oncology, The Royal Marsden Hospital NHS Foundation Trust, London, UK

**Keywords:** Anorectal melanoma, Wide local excision, Abdominoperineal resection, Morbidity, Mortality

## Abstract

**Purpose:**

Ano-uro-genital (AUG) Mucosal Melanoma UK guidelines recommended a less radical surgical strategy for anorectal melanoma (ARM) where possible. We report our experience of ARM consistent with that approach including clinical presentation, intervention undertaken and prognosis.

**Methods:**

We present a retrospective study of 15 consecutive patients with ARM surgically treated between November 2014 and April 2023. Patients were divided into the two surgery types: wide local excision (WLE, *n* = 9) and abdominoperineal resection (APR, *n* = 6). Data on demographics, diagnosis, treatment and oncological outcomes were assessed between the groups.

**Results:**

The mean age was 65.3 ± 17.4 years and 6 (40.0%) were female patients. Nine patients (60.0%) were diagnosed with stage I and six patients (40.0%) with stage II disease. R0 margins were achieved in all cases. The overall mean length of stay was lower following WLE compared to APR (2.6 ± 2.4 days versus 14.0 ± 9.8 days, *p* = 0.032). Two complications were observed in the WLE group compared to four complications after APR (*p* = 0.605). Five patients (55.5%) developed local/distant recurrence in the WLE group compared to three patients (50.0%) in the APR group (*p* = 0.707), with a median overall survival of 38.5 (12–83) months versus 26.5 (14–48) months, respectively.

**Conclusions:**

Achieving clear margins by the least radical fashion may have equivalent oncological outcomes to radical surgery, potentially reducing patient morbidity and preserving function. In our experience, the surgical management of ARM consistent with the ‘less is more’ approach adhering to AUG guidelines has acceptable outcomes.

## Introduction

Anorectal melanoma (ARM) is a rare disease accounting for less than 2% of all malignant melanomas [1]. Originating from melanocytes in the mucosa of the anorectal junction, approximately 60% of ARMs arise in the anal canal, with the remaining occurring within the rectum [2–6]. Associated symptoms are non-specific and these aggressive lesions are often mistaken for benign anorectal pathologies, such as haemorrhoids or adenomatous polyps, which may lead to delays in diagnosis and advanced disease at presentation. The prognosis of ARM is poor when compared with cutaneous melanoma, with reported 5-year survival rates ranging from 0 to 25% [7–13].

Despite advances in multi-modality therapy of melanoma, the improvement in ARM survival has been marginal [14, 15]. The preferred surgical intervention, either a conservative approach, in the form of wide local excision (WLE) to achieve narrow clear margins, or radical, in the form of abdominoperineal resection (APR), is debated. Historic series suggested that a radical approach offered improved locoregional control [11, 16], due to higher negative resection margins, and prevented disease dissemination by removing the source. However, radical resections are associated with considerable morbidity such as intra-abdominal abscess, stoma complications, incontinence, sexual dysfunction and subsequent psychosocial impact [17]. Recent studies have supported the use of WLE as a first-line approach in certain cases, describing no survival disadvantage and an improvement in quality of life when compared to APR [18–20]. However, given the low incidence of ARM and limited evidence available, there remains a lack of consensus on the optimal surgical strategy, as well as the required diagnostic imaging techniques and surveillance protocols for ARM.

In 2017, the Ano-uro-genital (AUG) Mucosal Melanoma UK guidelines were developed on the diagnosis, treatment and follow-up of patients with ARM using an evidence-based systematic approach [21–24] and is currently being updated. On behalf of the Guideline Development Group, Smith et al. [25] performed a systematic review and meta-analysis which recommended a less radical surgical approach for ARM where appropriate, particularly in the absence of clear evidence of oncological benefit from upfront radical surgery. We therefore describe our recent institutional experience of ARM consistent with this approach including clinical presentation, diagnosis, intervention undertaken, prognosis and surveillance protocols. This in turn will allow us to evaluate the ‘less is more’ strategy in line with the AUG guidelines in the context of a specialist centre.

## Methods

### Study Design and Participants

All patients with histologically proven ARM undergoing surgical intervention with curative intent at our institution, the Royal Marsden Hospital, London, were retrospectively analysed. After Institutional Review Board approval, 15 consecutive patients were identified between November 2014 and April 2023. All patients were discussed in an advanced specialist melanoma multidisciplinary tumour board meeting both pre- and postoperatively, with appropriate rectal cancer site-specific expertise. For the purpose of our analysis, patients were divided amongst the two surgery types, either WLE or APR. Patients were followed up intensively with a three-monthly examination under general anaesthesia, flexible sigmoidoscopy, computed tomography (CT) thorax, abdomen/pelvis or positron emission tomography (PET-CT) and magnetic resonance imaging (MRI) pelvis. This was followed by six-monthly clinical examinations, CT thorax, abdomen/pelvis and brain MRI if appropriate in accordance with the AUG guidelines [21].

Data including age, sex, American Society of Anesthesiologists (ASA) physical status classification system, presenting symptoms/signs, site of ARM, mean tumour thickness, tumour stage, neoadjuvant and adjuvant therapy, type of surgical intervention, length of stay, resection status and molecular profile of ARM were recorded. There is no TNM classification for ARM available, therefore the tumours were classified as stage I (localised disease), stage II (inguinal or pelvis lymph node metastasis) or stage III (distant metastasis) [26–28] in this study. Immunohistochemical stains were performed using S100 protein, Sox10, HMB-45, Melan-A, CD117 (c-KIT) and Vimentin. The main study outcome measures assessed were postoperative morbidity (categorised by Clavien-Dindo Classification, CDC) [29, 30]), mortality, overall survival (OS) and disease recurrence (local, nodal and/or distant). The OS was calculated from the date of diagnosis to the date of death or last follow-up.

This retrospective study was conducted using the Strengthening the Report of Observational Studies in Epidemiology (STROBE) guidelines [31].

### Statistical Analysis

The mean, median, standard deviation and range were calculated where applicable. Baseline characteristics and outcome variables were compared between WLE and APR groups using Mann-Whitney *U* test for continuous variables, and Fisher’s exact test for categorical variables where appropriate (*p* values of ≤ 0.05 were considered statistically significant). In addition, chi squared (*χ*^2^) testing was performed to determine whether there was a significant association between the type of surgery and other characteristics such as sex, site of tumour and medical therapy. Patients who initially had WLE then proceeded to APR were analysed as part of the APR group. Data was processed using JASP and R Studio software for descriptive and inferential statistics. The Kaplan-Meier method was used to plot survival curves and the survival between surgery types was compared with the log rank test.

## Results

### Patient Demographics and Tumour Characteristics

Fifteen patients with a confirmed tissue diagnosis of ARM that underwent surgery with curative intent were identified during the 9-year study period (Table 1). The mean age at the time of diagnosis was 65.3 ± 17.4 (range 34–95) years, with a male to female ratio of 1.5:1. The most common presenting complaint was bleeding per rectum (53.0%) followed by prolapsing sensation (20.0%) and anal discomfort (13.3%). Most patients (86.7%) had an initial incorrect diagnosis, including haemorrhoids, rectal polyp or a benign mass. The tumours were located at the anal canal (73.3%), perianal (20.0%) or anorectal region (6.7%). Nine patients (60.0%) were diagnosed with stage I and six patients (40.0%) with stage II disease. Immunohistochemically, diffuse protein expression of Melan-A, S100 protein and HMB-45 was seen in 12 (80.0%), 9 (60.0%) and 6 (40.0%) cases, respectively. Six gene (40.0%) mutations were detected in total. BRAF, NRAS and Kit mutations were reported in 0 (0.0%), 2 (13.3%) and 2 (13.3%) of cases, respectively.

**Table 1:** Tumour characteristics and oncological outcomes of fifteen patients with anorectal melanoma

**Patient and tumour characteristic**	**Patients (*n*** **= 15)**
**Mean age, years (range)**	65.3 ± 17.4 (34–95)
**Sex, *n* (%)**	
Male	9 (60.0)
Female	6 (40.0)
**ASA, *n* (%)**	
I	2 (13.3)
II	10 (66.7)
III	3 (20.0)
**Site of tumour, *n* (%)**	
Anal canal	11 (73.3)
Perianal	3 (20.0)
Anorectal	1 (6.7)
**Mean tumour size (mm)**	17.1 ± 16.6
**Tumour stage, *n* (%)**	
I	9 (60.0)
II	6 (40.0)
III	0 (0.0)
**Immunohistochemical stain positivity, *n* (%)**	
Melan-A	12 (80.0)
S100 protein	9 (60.0)
HMB-45	6 (40.0)
Sox10	4 (26.7)
CD117 (c-KIT)	1 (6.7)
Vimentin	1 (6.7)
**Surgical approach, *n* (%)**	
Wide local excision	9 (60.0)
Abdominoperineal resection	6 (40.0)
**Medical therapy, *n* (%)**	
Preoperative immunotherapy	2 (13.3)
Preoperative radiotherapy	2 (13.3)
Adjuvant immunotherapy	7 (46.7)
Adjuvant chemotherapy	1 (6.7)
Adjuvant BRAF and MEK inhibitor	1 (6.7)
**Recurrence, *n* (%)**	8 (53.3)
Local	2 (13.3)
Nodal	4 (26.6)
Distant	5 (33.3)
**Median time to recurrence, months (range)**	7.7 (0.7–16.9)
**Median overall survival, months (range)**	30.0 (12.0–83.0)
**Mortality from ARM, *n* (%)**	6 (40.0)

*ARM *anorectal melanoma,* ASA* American Society of Anesthesiologists physical status classification system

### Surgical Management of ARM and Oncological Outcomes

Nine patients (60.0%) underwent WLE and five patients (40.0%) had APR surgery. None of the patients were deemed suitable for anterior resection. R0 margins (microscopically clear > 1 mm) were achieved in all 15 cases. In the WLE group, R0 margins were achieved on the first excision in eight cases (88.9%) and on the second excision in one case (11.1%). A normal sphincter function was reported in all WLE cases. Neoadjuvant and adjuvant immunotherapy was administered in two (13.3%) and seven patients (46.6%) respectively, in the form of ipilimumab and nivolumab. Preoperative radiotherapy was given in two cases (13.3%) and adjuvant chemotherapy in one case (6.7%). Adjuvant targeted therapies, dabrafenib and trametinib, were administered to one patient (6.7%).

After a mean follow-up period of 34.3 months, recurrence was identified in eight patients (53.3%), of which local and distant recurrence occurred in two (13.3%) and five cases (33.3%) respectively. Distant metastatic sites included lung, liver, mediastinal and hilar regions. The following investigations identified the site of recurrence: CT thorax, abdomen/pelvis (*n* = 3, 50.0%), clinical examination and rectal MRI (*n* = 3, 50.0%), PET-CT (*n* = 1, 16.7%) and flexible sigmoidoscopy with biopsies (*n* = 1, 16.7%). The overall median time to recurrence was 7.7 (0.7–16.9) months and the median OS was 30.0 (12–83) months. During the follow-up period, seven patients (46.7%) had no evidence of disease at the time of assessment (Table 2). Two patients (13.3%) were alive with evidence of disease and one patient (6.7%) died of complications including worsening heart failure and metastatic prostate cancer. Five patients (33.0%) died of the disease, four of which initially had stage II disease.

**Table 2:** Disease status and survival of patients with anorectal melanoma according to tumour staging

**Stage**	**No evidence of disease,** ***n*** **(%)**	**Alive with disease,** ***n*** **(%)**	**Died of disease,** ***n*** **(%)**	**Died of complications,** ***n*** **(%)**
**I**	7 (46.7)	0 (0.0)	1 (6.7)	1 (6.7)
**II**	0 (0.0)	2 (13.3)	4 (26.7)	0 (0.0)
**III**	0 (0.0)	0 (0.0)	0 (0.0)	0 (0.0)
**Total**	7 (46.7)	2 (13.3)	5 (33.3)	1 (6.7)

Stage I is defined as localised disease, Stage II as inguinal or pelvis lymph node metastasis and Stage III as distant metastasis

### Wide Local Excision Versus Abdominoperineal Resection

The patients in this study were divided into two groups: WLE (*n* = 9, 60%) and APR group (*n* = 6, 40%) (Table 3). There were no differences in age or ASA grade between the two groups. The mean tumour size was larger in the APR group (23.0 ± 22.3 mm) compared to the WLE group (13.5 ± 12.4 mm), however this was not statistically significant (*p* = 0.339). Regarding the molecular profile, there were two NRAS (22.2%) and Kit (22.2%) mutations reported in the WLE group. In the APR group, there were two mutations (33.3%) reported; however, there were no mutations in specifically BRAF, NRAS or Kit genes (Table 4).

**Table 3:** Tumour characteristics, postoperative and oncological outcomes of patients undergoing wide local excision and abdominoperineal resection

**Patient and tumour characteristic**	**WLE (*n* = 9)**	**APR (*n* = 6)**	***p*** **value**
**Mean age, years (range)**	65.0 ± 15.4 (36–84)	65.8 ± 21.6 (34–95)	0.931
**Sex, *n* (%)**			
Male	4 (44.4)	5 (83.3)	0.133
Female	5 (55.6)	1 (16.7)	
**ASA, *n* (%)**			
I	1 (11.1)	1 (16.7)	
II	7 (77.8)	3 (50.0)	0.372
III	1 (11.1)	2 (33.3)	
**Site of tumour, *n* (%)**			
Anal canal	6 (66.7)	5 (83.3)	
Perianal	3 (33.3)	0 (0.0)	0.139
Anorectal	0 (0.0)	1 (16.7)	
**Mean tumour size, mm**	13.5 ± 12.4	23.0 ± 22.3	0.339
**Tumour stage, *n* (%)**			
I	3 (33.3)	5 (83.3)	
II	6 (66.7)	1 (16.7)	0.121
III	0 (0.0)	0 (0.0)	
**Mean number of surgical procedures**	2.14 ± 1.7	1.57 ± 0.8	0.439
**Overall mean length of stay (days)**	2.6 ± 2.4	14.0 ± 9.8	0.032^a^
**Postoperative complications, *n* (%)**	2 (22.2)	4 (66.7)	
CDC I	1 (11.1)	0 (0)	
CDC II	1 (11.1)	3 (50.0)	0.605
CDC III	0 (0.0)	1 (16.7)	
CDC IV	0 (0.0)	0 (0.0)	
CDC V	0 (0.0)	0 (0.0)	
**Medical therapy, *n* (%)**			
Preoperative immunotherapy	1 (11.1)	1 (16.7)	
Preoperative radiotherapy	1 (11.1)	1 (16.7)	0.667
Adjuvant immunotherapy	4 (44.4)	3 (50.0)	
Adjuvant chemotherapy	1 (11.1)	0 (0.0)	
Adjuvant BRAF and MEK inhibitor	1 (11.1)	0 (0.0)	
**Recurrence, *n* (%)**	5 (55.5)	3 (50.0)	
Local	2 (11.1)	0 (0.0)	
Nodal	3 (33.3)	1 (16.6)	0.707
Distant	3 (33.3)	2 (33.3)	
**Median time to recurrence, days (range)**	7.0 (0.7–16.9)	9.0 (4.9–10.9)	0.786
**Median overall survival, months (range)**	38.5 (12.0–83.0)	26.5 (14.0–48.0)	0.145
**Mortality from ARM, *n* (%)**	4 (44.4%)	2 (33.3%)	0.4

*ARM *anorectal melanoma*, ASA* American Society of Anesthesiologists physical status classification system, *APR* abdominoperineal resection, *CDC* Clavien-Dindo Classification, *WLE* wide local excision

^a^Statistically significant

**Table 4:** Molecular profile of patients undergoing wide local excision and abdominoperineal resection

**Molecular analysis**	**BRAF gene mutation,** ***n*** **(%)**	**NRAS gene mutation,** ***n*** **(%)**	**Kit gene mutation,** ***n*** **(%)**	**Other gene mutation,** ***n*** **(%)**
**WLE, *n* = 9**				
Detected	0 (0.0)	2 (22.2)	2 (22.2)	0 (0.0)
Not detected	6 (66.7)	4 (44.4)	2 (22.2)	6 (66.7)
Unknown	3 (33.3)	3 (33.3)	5 (55.6)	3 (33.3)
**APR, *n* = 6**				
Detected	0 (0.0)	0 (0.0)	0 (0.0)	2 (33.3)
Not detected	5 (83.3)	5 (83.3)	6 (100.0)	4 (66.7)
Unknown	1 (16.7)	1 (16.7)	0 (0.0)	0 (0.0)
**Total**				
Detected	0 (0.0)	2 (13.3)	2 (13.3)	2 (13.3)
Not detected	11 (73.3)	9 (60.0)	8 (53.3)	10 (66.7)
Unknown	4 (26.7)	4 (26.7)	5 (33.3)	3 (20.0)

*APR* abdominoperineal resection, *WLE* wide local excision

The six patients in the WLE group underwent more frequent surgical procedures (examination under anaesthesia, flexible sigmoidoscopy or inguinal lymph node dissection) with a mean of 2.14 ± 1.7 procedures compared to 1.57 ± 0.8 procedures in the APR group (*p* = 0.439). Five patients (55.5%) in the WLE group underwent inguinal lymph node dissection either at the same time as WLE or after. Five patients (83.3%) in the APR group went directly for radical surgery, with one patient undergoing several prior WLE procedures. The overall mean length of stay was significantly lower following WLE compared to APR (2.6 ± 2.4 days versus 14.0 ± 9.8 days, *p* = 0.032). There were two (22.2%) postoperative complications in the WLE group related to inguinal lymph node dissection, including wound dehiscence (CDC I) and lymphoedema (CDC II). This compared to four (66.6%) complications in the APR group (*p* = 0.605), of which three (50.0%) were graded as CDC II (chest, wound and urinary tract infection managed with intravenous antibiotics) and one (16.7%) graded as CDC III (pelvic collection requiring radiological drainage).

**Fig. 1:**
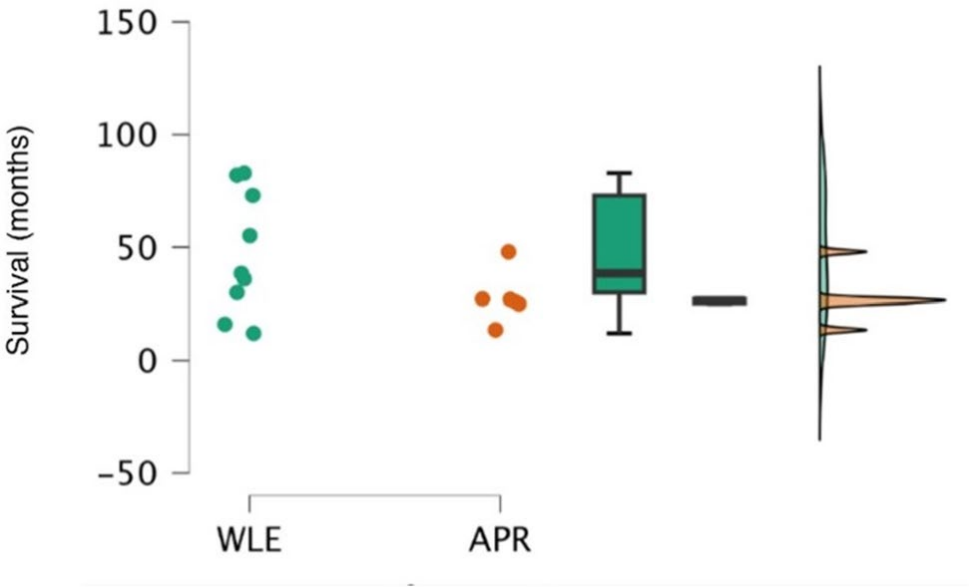
Comparison of survival between wide local excision (*n* = 9) and abdominoperineal resection (*n* = 6) groups in patients with anorectal melanoma. *APR* abdominoperineal resection; *WLE* wide local excision

**Fig. 2:**
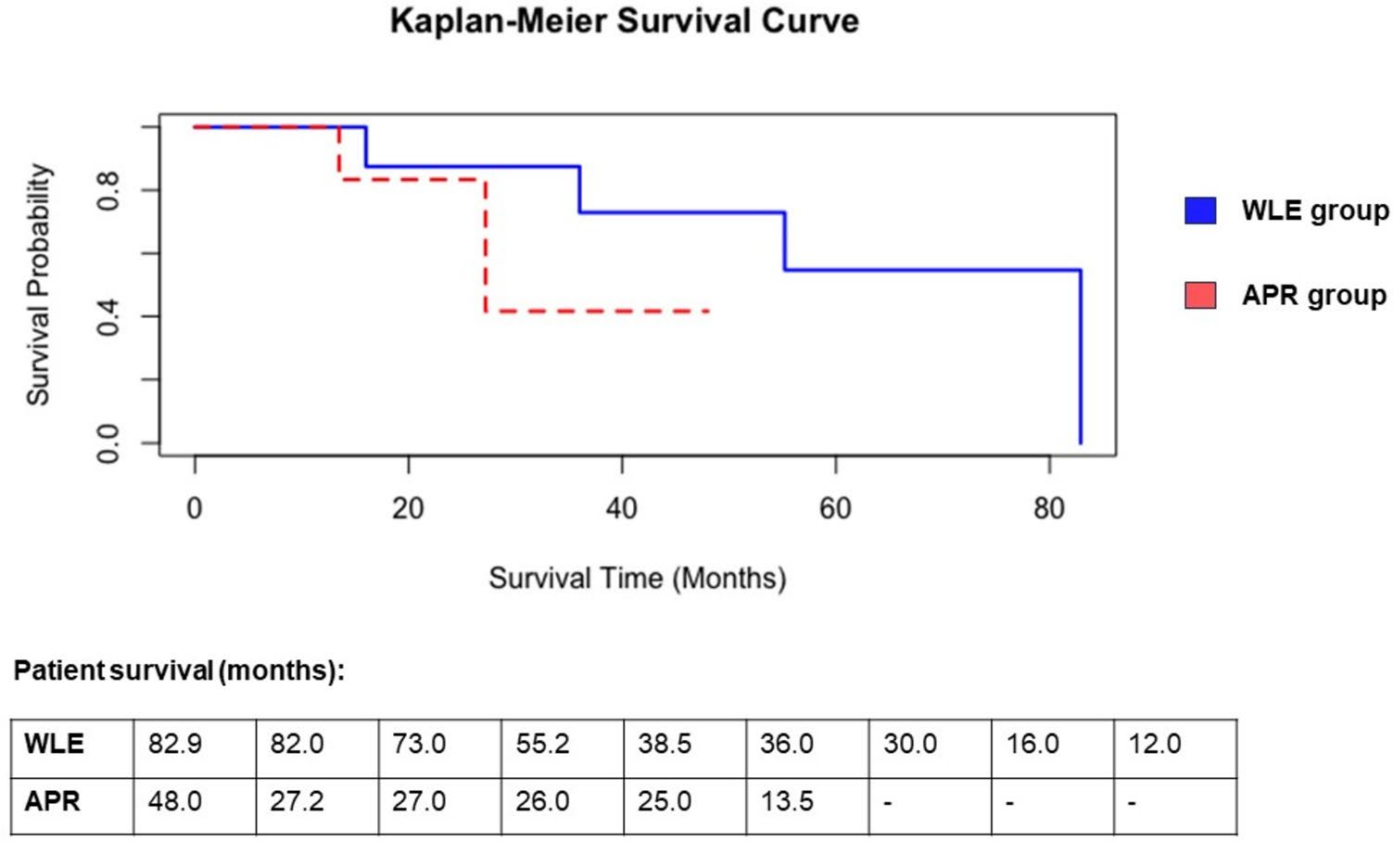
Kaplan-Meier survival curves for wide local excision (*n* = 9) and abdominoperineal resection (*n* = 6) groups in patients with anorectal melanoma. The overall median survival was 38.5 months versus 26.5 months, respectively (*p* = 0.145). *APR* abdominoperineal resection, *WLE* wide local excision.

In terms of oncological outcomes, recurrence of disease was identified in three patients (50.0%) in the WLE group and five patients (55.5%) in the APR group (*p* = 0.707). Of these recurrences, distant disease was seen in a third of cases in both groups. The median time to recurrence was 7.0 (0.7–16.9) months after WLE and 9.0 (4.9–10.9) months following APR (*p* = 0.786). The median OS was 38.5 (12–83) months in the WLE group compared to 26.5 (14–48) months in the APR group (*p* = 0.145) (Fig. 1). When assessing the Kaplan-Meier survival probability curves, there is an observational survival benefit for the WLE group, particularly between 24 and 48 months after surgery; however, this is not significant for survival from the date of diagnosis (*p* = 0.400) and when adjusted to survival from the date of surgery (*p* = 0.300) (Fig. 2).

## Discussion

ARM is an aggressive disease with nearly half the patients in our study presenting with stage II disease, reflecting its rare incidence and non-specific presentation resulting in misdiagnoses. Immunohistochemical markers, for example Melan-A, S-100 protein and HMB-45, may be used to aid the diagnosis. However, the frequency of BRAF, NRAS and Kit mutations in ARM is lower than that of cutaneous melanoma [32–36] which can also be inferred by our study. Although it is known that women are more likely to be diagnosed with ARM than men [37], we found a higher proportion of men affected by ARM in our study. The stage of disease at presentation may have been a contributing factor to the high recurrence rate (53.3%) and poor mortality rate (40.0%) observed during the follow-up period. The WLE group had a median OS of 38.5 months compared to 26.5 months in the APR group, despite a greater proportion of patients presenting with stage II disease in the WLE group. There was no distinct survival benefit accrued over clear margins associated with WLE or APR in our series, which is comparable to previous retrospective studies assessing the surgical management of ARM [7, 8, 10, 11, 16, 18, 27, 37–40].

The AUG guidelines produced by the Guideline Development Group on behalf of Melanoma Focus [41], has since been accredited by the National Institute for Health and Care Excellence. This coincided with a meta-analysis [25] which found that there was no difference in local disease control or survival with radical surgery compared to conservative surgery in ARM. It was therefore recommended that WLE with regular surveillance should be the primary strategy in most cases, unless sphincter function is likely to be compromised from a WLE, then APR should be considered.

However, due to the rarity of ARM and the lack of prospective studies, the choice of surgical treatment remains controversial for ARM. Some authors recommend radical surgery such as APR [11, 13, 16] as it may control lymphatic spread and lead to larger negative margins and therefore a potentially lower recurrence rate, whilst other studies have proposed a more conservative approach if negative margins can be achieved by WLE [9, 14, 19, 42, 43]. Our experience suggests that this treatment paradigm of a less radical approach, consistent with the AUG guidelines, has acceptable oncological outcomes, with lower morbidity and perioperative complications. Radical surgery did not necessarily improve survival outcomes and can lead to relatively significant morbidity in patients.

The precise role of neoadjuvant and adjuvant medical therapy remains unclear [44, 45]. Patients may be amenable to targeted therapy with imatinib for c-KIT and BRAF mutations, but the mutations are relatively infrequent. The benefit of immunotherapy is clearly established in cutaneous melanoma [46]; however, the response appears to be reduced in ARM. Nevertheless, in a recent study by Ho et al. [47], neoadjuvant immunotherapy was deemed a feasible and safe approach for resectable mucosal melanoma. Nineteen out of 36 patients had ARM and the majority of these patients received PD1 and CTLA-4 inhibitors. The 3-year recurrence-free survival rate was 29% and the 3-year OS rate was 55% which compares favourably to other studies [48].

Increasingly, single agent PD1 is being recognised as the de facto standard of care in the adjuvant treatment of melanoma for patients with BRAF wild-type tumours. In the Checkmate 238 study, nivolumab showed sustained recurrence-free survival benefit in resected stage IIIB-C or IV melanoma, of which 29 patients had mucosal melanoma [49]. It has recently been suggested that adjuvant chemotherapy (temozolomide plus cisplatin) may have recurrence-free survival, distant metastasis-free survival and OS compared to a PD1 inhibitor (toripalimab) in resected mucosal melanoma [50]. The recurrence-free survival with chemotherapy and PD1 inhibitor was found to be 28.2 versus 12.0 months, the distant metastasis-free survival was 42.0 versus 19.0 months and the OS was 93.4 versus 39.3 months, respectively.

There are two further studies which may help to inform our approach in the future. The SALVO trial (NCT03241186) involves a single-arm trial of ipilimumab and nivolumab as adjuvant therapy for resected mucosal melanoma. Thirty-five patients over four years have been recruited thus far, of which 11 patients had ARM. The reported 2-year recurrence-free survival and OS was 37% and 68%, respectively. This compared to 2-year recurrence-free survival rates of 0% in the study by Lian et al. [48]. Neoadjuvant Pembrolizumab and Lenvatinib for Mucosal Melanoma (NeoPeLemm) will be a multicentre, open label, phase II trial (NCT05545969) that will aim to determine the response to neoadjuvant pembrolizumab and lenvatinib followed by adjuvant pembrolizumab in resectable mucosal melanoma.

### Proposed Recommendations for the Management of ARM

The AUG guidelines are supported by the findings of this retrospective study, and as such, we have been able to confirm and propose further clinical recommendations. To begin with, each individual case of ARM should be discussed in a specialist melanoma multidisciplinary team (MDT) meeting with input from the colorectal cancer site-specific expertise. Staging investigations should include digital rectal examination, palpation of inguinal lymph nodes, examination under anaesthesia, flexible sigmoidoscopy, CT thorax, abdomen/pelvis and MRI pelvis. If APR is being considered, a PET-CT scan should be performed to help define the appropriate surgical strategy.

Management of ARM should be carried out only in centres regularly performing complex anorectal surgery and managing complex melanoma within a specialist melanoma MDT. The patient’s baseline anorectal function should be assessed, as well as the resectability based on staging investigations and a pathology review of molecular profiling. The ultimate aim is to achieve an R0 margin in the least radical fashion with WLE where possible. WLE should be repeated as required and lymph node biopsy should only be performed if it directs adjuvant treatment. APR should be reserved for lesions that cannot be removed by local excision or for salvage surgery in the case of isolated recurrence, as well as when there is evidence of anal sphincter invasion or mesorectal lymphatic involvement. Patient preferences and quality of life is also essential in this decision-making process. After MDT discussion, if the ARM is deemed irresectable or present with distant metastatic disease, other management options should be considered including systemic therapy such as combination immunotherapy, radiotherapy or palliative surgery. Some authors have reported that radiotherapy can have a beneficial effect on ARM [42, 51] but further studies are required to confirm this and understand the fractionation and timing in relation to surgery. Following treatment, ARM requires an intensive surveillance protocol which were set out by Smith et al. [25] and followed in this retrospective study.

### Limitations

There are important limitations that must be considered when interpreting the study findings. This study was retrospective in nature, with a small, highly selected sample over a 9-year period. There were no patients who presented with stage III, which may have led to a generally higher survival compared to other series, and likely reflects referral bias to our centre. Detailed information on patient reported outcomes and quality of life was also missing. In addition, patients were managed with different treatment regimens rendering it difficult to make robust comparisons between the two surgery types. Although difficult due to the rarity of ARM, prospective studies, with a larger sample size, comparing WLE and APR is required to make a thorough assessment of the efficacy and safety of these approaches, as well as patient reported outcomes and quality of life.

## Conclusions

In our experience, radical surgery did not improve survival or recurrence outcomes and is associated with significant morbidity which supports the AUG Mucosal Melanoma UK national guidelines. Achieving clear margins, in the least radical fashion, is important for recurrence-free and OS whilst simultaneously maintaining overall function. WLE with regular surveillance should therefore be the primary strategy in most patients if feasible. Specialist care with expert melanoma and site-specific surgical care is essential, along with an intensive surveillance regimen and close cooperative MDT care. Further research is required to determine the precise role and timing of systemic anti-cancer therapies and radiotherapy in ARM.

## Data Availability

The datasets generated during and/or analysed during the current study are available on request from the corresponding author.
